# Personality assessment usage and mental health among Chinese adolescents: A sequential mediation model of the Barnum effect and ego identity

**DOI:** 10.3389/fpsyg.2023.1097068

**Published:** 2023-02-02

**Authors:** Jie Hua, Yi-Xin Zhou

**Affiliations:** School of Journalism and Communication, Nanjing University, Nanjing, China

**Keywords:** personality assessment, Barnum effect, ego identity, anxiety, subjective well-being, depression, MBTI, adolescent mental health

## Abstract

**Introduction:**

Adolescence is a crucial period for establishing ego identity and becoming a social individual. However, numerous adolescents suffer from mental health problems, especially after the conditions surrounding the COVID-19 outbreak. Personality assessments are often used when adolescents look for psychological self-help services. However, the meaning and mechanism of these personality assessments remain unknown. Taking the increasingly popular MBTI personality assessment as an entry point, the current study examined the potential sequential mediation relationship of Barnum effect – ego identity on the link between personality assessment usage and mental health.

**Methods:**

The current study surveyed 308 Chinese high school students, including 109 males and 199 females. MBTI use, Barnum effect, ego-identity, and mental health (subjective well-being, depression, and anxiety) were measured by seven questionnaires, respectively. Sequential mediation models were constructed to analyze the relationship.

**Results:**

The results indicate that the Barnum effect and ego identity together function as a sequential mediation path between personality assessment use and teenagers’ mental health, including subjective well-being, depression, and anxiety. Specifically, a higher level of MBTI use triggers a stronger Barnum effect. The Barnum effect then promotes adolescents’ ego identity, ultimately increasing subjective well-being levels and reducing anxiety and depression.

**Discussion:**

Our findings suggest that by properly using personality assessment and stimulating the Barnum effect, we can enhance adolescents’ mental health. The theoretical and practical implications of our findings are discussed.

## 1. Introduction

Adolescence is a crucial period for establishing ego identity and becoming a social being. However, under the conflicts of society, culture, and self, adolescents at this stage typically fall into “identity crises” (Erikson, 1994). Data show that the mental health situation of adolescents worldwide is not optimistic. Approximately 20% were diagnosed with mental health problems, while the detection rate of depression and anxiety was close to 30% (Centers for Disease Control and Prevention, 2019; Chen et al., 2020). The outbreak of COVID-19 has made the situation worse. During the pandemic, the severity and incidence of psychological problems among adolescents have increased (Zhang et al., 2020; Ford et al., 2021; Jones et al., 2021; Panchal et al., 2021). It is urgent to design effective and feasible psychological interventions to enhance adolescent mental health.

When encountering psychological problems, adolescents prefer seeking self-help information support (Rickwood et al., 2007). Among them, personality assessments are favored, including some low-scientific ones (Archer et al., 1991; Das et al., 2022). However, whether and how personality assessments affect adolescent mental health remains unknown. Recently, one of the personality assessments, the Myers-Briggs Type Indicator (MBTI), has received a dramatic increase in attention on Chinese Social Networking Sites (SNSs), especially among adolescents (Baidu Index, 2022). On Weibo, the number of readings related to “MBTI” and “MBTI memes” exceeded 6 billion, with more than 2 million discussions. The MBTI is a personality assessment based initially on Jungian psychological types. However, its reliability and validity do not meet scientific standards (Pittenger, 2005). Therefore, the MBTI can serve as an entry point in our exploration.

The Barnum effect, the tendency of people to accept general personality descriptions as their own characteristics (Snyder et al., 1977), provides a possible explanation for how these low-validity personality assessments contribute to adolescent mental health. A few prior studies have suggested that the Barnum effect may play a role in the relationship between personality assessment and ego identity, which is strongly associated with mental health. However, such a relationship has yet to be empirically examined.

This study seeks to explore whether the Barnum effect triggered by the MBTI can help to enhance adolescents’ ego identity and whether the relationship between MBTI use and mental health (subjective well-being, depression, and anxiety) among Chinese adolescents can be sequentially mediated by the Barnum effect and ego identity.

## 2. Literature review

### 2.1. Personality assessment and mental health

Personality describes a unique pattern of a person’s thoughts and behaviors that distinguishes one person from another (Mischel et al., 2007; Eysenck, 2014). Personality assessments are methods to quantify personality (Hannay et al., 2010). As it matured in the 20th century, personality assessments gradually became essential instruments in the professional world for understanding human characteristics.

One of the ancient personality assessments is astrology. Certain astrological beliefs classify people into 12 astrological types based on an individual’s date of birth, believing that each type has a different personality. To this today, Astrology is popular and influential worldwide (Eurobarometer, 2005; Besley and Hill, 2020). However, astrology is regarded as unscientific by the academic community (Lu et al., 2020).

Another prevalent personality assessment among the general public today is the Myers–Briggs Type Indicator (MBTI; Myers et al., 1985). The MBTI model divides personality differences into four dimensions: (1) the perceptual orientation of an individual, Extraversion–Introversion; (2) their preferred way of obtaining information, Sensing–Intuition; (3) their preferred method of making decisions, Thinking–Feeling; and (4) their way of dealing with the external world, Judgment–Perception (Pittenger, 2005). Through the combination of four dimensions, the MBTI classifies people into 16 personality types (e.g., INFJ, ESTP, etc.; Pittenger, 2005). Since its inception as a formal instrument in 1942 (as initially used for selection and training by the United States military), the MBTI has extended and maintained worldwide influence (Furnham, 1996). The reliability and validity of the MBTI have also been questioned. Theoretically, it classifies people into two opposite types in each dimension (McCrae and Costa, 1989), which obey the continuous quasi-normal distribution of personality (Hicks, 1984). Empirically, the retest reliability of the MBTI did not exceed 0.6 (Levy et al., 1972; Johnson, 1992; Tsuzuki and Matsui, 1997). Its construct and external validity were also lower than the standard (Pittenger, 2005).

With the fact that the MBTI and astrology are low-scientific but prevalent, more is needed to know about the ensuing social significance of these personality assessments provided to individuals. Rarely have studies investigated the relationship between personality assessments and mental health. Nevertheless, some evidence supports this potential link. Firstly, data showed that adolescents sought support from some personality assessments when encountering psychological problems (Archer et al., 1991; Rickwood et al., 2007; Das et al., 2022). This indicates positive implications of personality assessments. Secondly, researchers suggested that personality assessments in consultations are beneficial and therapeutic (Halperin and Snyder, 1979; Lee and Park, 2014). The results of psychological tests can predict a person’s ego strength and indicate primary ways to handle their life (Mosak and Gushurst, 2018). Thirdly, when looking into popular culture, astrology information on SNSs was found to possibly help alleviate stress from uncertainty (Lopez et al., 2021).

In sum, personality assessment use may be positively associated with individuals’ subjective well-being and negatively associated with depression and anxiety.

*H1*: MBTI use is positively related to (a) subjective well-being and negatively related to (b) depression and (c) anxiety.

### 2.2. Ego identity as a mediator

The term “ego identity” (“self-identity” has been used in some studies for the same concept) originated from Erikson’s (1994) formulation of identity development. It refers to the essential stage in which teenagers transform their childhood identifications into coherent and personally meaningful identities (Soenens and Vansteenkiste, 2011). Marcia et al. (2012) operationally define ego identity into two dimensions: exploration and commitment. The former refers to the period of exploring various possibilities of personal identity and ideological beliefs. The latter describes making a firm decision in such areas and investing in ideal activities. By combining these two dimensions, ego identity can be classified into four statuses: (1) achievement (strong commitment with full exploration), (2) foreclosure (strong commitment without exploration), (3) moratorium (active exploration without commitment), and (4) diffusion (the absence of both commitment and exploration; Marcia et al., 2012).

Although the determinants of adolescents’ ego identity were well-studied, limited studies have investigated the influence of external personality-related information on ego identity. However, personality assessment usage is likely to shape adolescent ego identity. Firstly, information from the social world is one of the most important sources for people to obtain self-perception (Brown, 2014). In daily life, people cannot observe or reflect on themselves as outsiders in every moment. They need to rely on external information to construct a basic self-cognition. According to Erikson (1994), ego identity is an increasingly coherent self-cognition under psychodynamics, personal life history, self-belief, and other factors. Therefore, self-relevant information from the outside world, such as personality assessment feedback, may influence ego identity through the cognitive process. Secondly, the topological model of the MBTI conforms to the workings of the human brain (Tversky and Kahneman, 1974). Everyone can be classified into one of 16 types, which enables individuals to make some easy-to-understand interpretation of their inner selves, even though the types are overly generalized.

Empirically, Lee and Park’s (2014) experiment demonstrated that adolescents who participated in astrology counseling developed higher self-awareness than non-astrology counseling adolescents. Another experiment using astrology, German, and psychology courses as materials also found a causal relationship between astrology and self-concept (Lillqvist and Lindeman, 1998). According to Guo and Che’s (2004) and Makros and McCabe’s (2001) research, there is an overlap between self-identity and other concepts related to the self. Therefore, the findings discussed above can be extended to the study of ego identity. We hypothesize that the usage of personality assessments such as MBTI influences adolescents’ ego identity.

*H2*: MBTI use is positively related to ego identity.

Researchers have found a strong connection between ego identity and mental health. According to Erikson (1994) and Marcia et al. (2012)’s theory, the establishment of ego identity contributes to adolescents’ physical and mental health, promotes their better adaptation to society, and has far-reaching implications for their self-actualization and future development. In an empirical study, Waterman (2007) explored the relationships among the four identity statuses and the three types of well-being states. He found that undergraduates with an achievement status (high commitment and high exploration) had higher levels of well-being. Other statuses, due to deficits in exploration or commitment, showed lower levels of well-being. Many studies have also demonstrated that self-identity helps to enhance individual well-being (Hofer et al., 2007; Cakir, 2014). In addition, a survey by Schwartz et al. (2011) explored the negative dimensions of well-being. They found that individuals with a diffuse identity showed the highest levels of depression and anxiety, while individuals with an achievement identity showed the lowest.

In summary, personality assessments can provide insight into ego identity in adolescents, and there are connections between ego identity and adolescent mental health.

*H3*: Ego identity mediates the relationship between MBTI use and mental health [(a) subjective well-being, (b) depression, and (c) anxiety].

### 2.3. The Barnum effect and ego identity as sequential mediators

The “Barnum effect” refers to a psychological phenomenon in which people easily accept general and ambiguous personality interpretations and believe they accurately express their unique characteristics. This cognitive trend was initially detected in astrology (Forer, 1949) and later found in various low-scientific personality assessments, such as graphology and MBTI (Tyson, 1982a,b).

Personality assessment use has a strong association with the Barnum effect. Firstly, apart from individual traits such as neuroticism (Fichten and Sunerton, 1983) that would increase the Barnum effect, information characteristics also play a role. The focal information characteristics include the positivity of the content (Mosher, 1965; Halperin et al., 1976), the scientific nature of the source, and the professionalism of the procedure (Dickson and Kelly, 1985). Additionally, the Barnum effect works due to the subjective validation effect and the flattery effect. The former refers to the phenomenon in which a person feels a description is appropriate because it is personally meaningful or crucial to them (Forer, 1949). The latter describes the tendency that most people are willing to believe things that make them appear more positive (Wiseman, 2007). These information characteristics and underlying processes can be found in self-related personality assessments such as astrology and MBTI. Thus, despite these personality assessments’ low validity, people continue to accept these “Barnum personality feedbacks” as an accurate description of their traits (Furnham and Schofield, 1987).

Some evidence indicates that the Barnum effect can contribute to individuals’ ego identity. Self-verification theory suggests that people would like to obtain objective, accurate and diagnostic information to reduce uncertainty about themselves. Once such certainty perceptions are formed, people will strive to justify them (Swann and Read, 1981). The Barnum effect describes the recognition and belief of feedback about people’s personalities. According to the self-verification theory, this recognition and belief can further enhance people’s prediction and control over reality, which in turn, contributes to the formation of a stable self-concept (Swann and Read, 1981). In addition, the Barnum statements are mostly positive, stimulating individuals’ recognition for creating a “positive illusion” about the self. This positive illusion acts as a cognitive filter that selects and organizes self-relevant information. Such a process helps to achieve self-service and self-enhancement (Taylor and Brown, 1999).

Several studies also provided empirical support for the potential mediating role of the Barnum effect in the association between personality assessment and ego identity. Halperin and Snyder (1979) demonstrated that the Barnum effect could be utilized to enhance clients’ personal expectations in the potential of self-change. The expectations would then actually facilitate therapeutic improvement. Lillqvist and Lindeman’s (1998) found students taking astrology courses had higher certainty about their self-concept than students taking German and psychology courses. The Barnum effect was considered a possible explanatory factor. They also raised the possibility that astrological information affects individuals’ well-being through this path. Recently, Lopez et al. (2021) found significant associations among exposure to astrology information, the Barnum effect, and individual stress levels.

Collectively, these studies outline the potential role of the Barnum effect in helping to enhance an individual’s ego identity. Together, we hypothesized that there would be a significant sequential mediation path from MBTI use, to the acceptability of the Barnum effect, to ego identity, and then to adolescents’ subjective well-being, depression, and anxiety.

*H4*: The Barnum effect and ego identity mediate the relationship between MBTI use and mental health ([a] subjective well-being, [b] depression, and [c] anxiety) sequentially.

**Figure 1:**
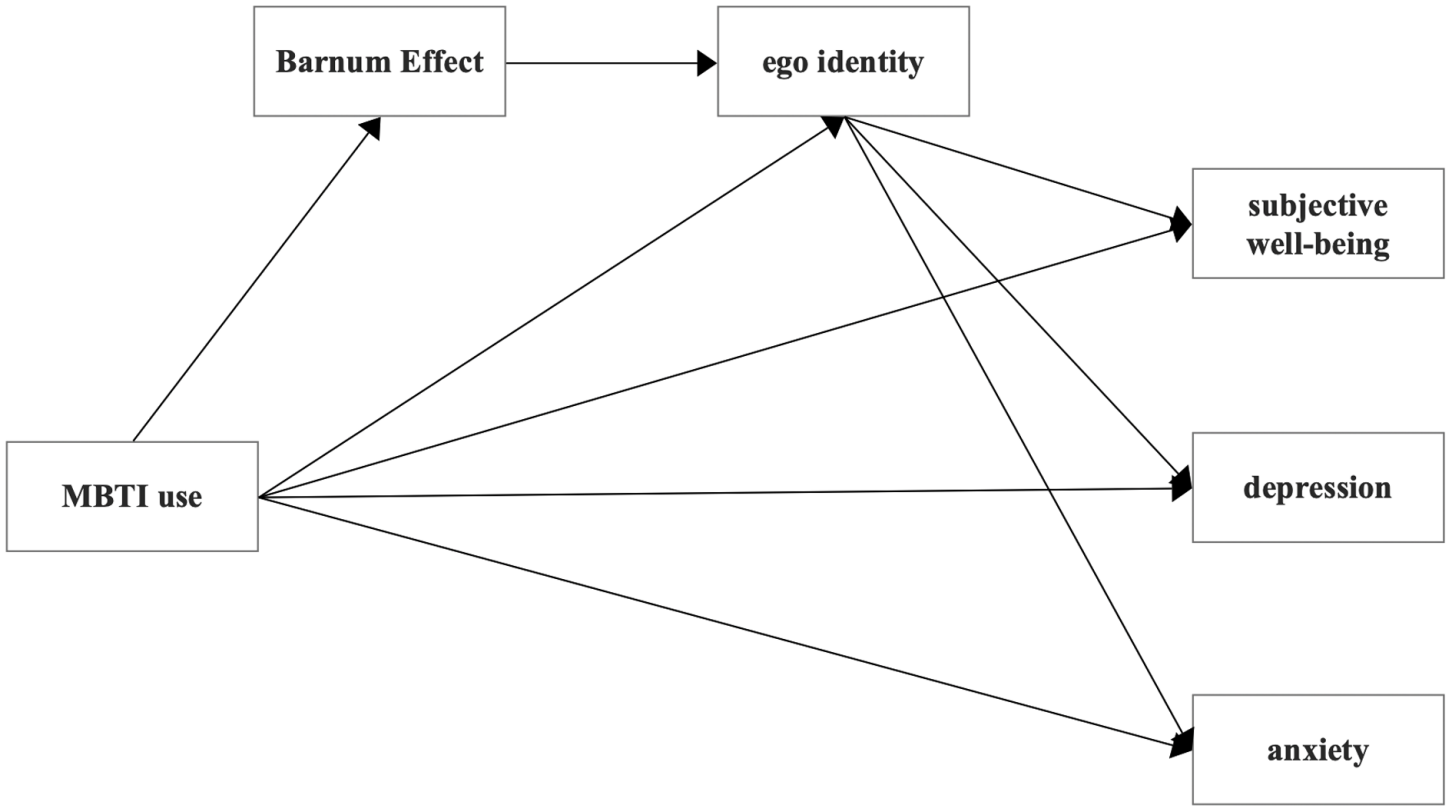
Diagrams of the proposed hypotheses.

The conceptual model of this study is illustrated in Figure 1.

## 3. Methods

### 3.1. Participants

Before the survey, we conducted Monte Carlo Power Analysis for Indirect Effects application (Schoemann et al., 2017) to estimate the sample size. The minimum sample size was 273 to reach power.90, assuming correlations of *r* = 0.30 (SD = 0.10) among the independent variable, the mediators, and the dependent variables. The current study collected a sample of 403 high school students, which is adequate to produce reliable results. Participant recruitment and survey conduction are both done *via* Wenjuanxing,^1^ a widely known and applied Chinese online questionnaire platform. After completing the online questionnaire, which took about 5–10 min, each participant received 27 Chinese yuan as compensation.

Excluding 95 participants who failed our attention check question or selected “have not heard of MBTI before,” a total of 308 participants qualified for the study. The final sample consisted of 109 males (35.4%) and 199 females (64.6%). Their ages were: 15 (4.9%), 16 (17.2%), 17 (37%), and 18 (40.9%); the average age was 17.19 (SD = 0.871). The subjective social class of the sample roughly approximated a normal distribution. The descriptive statistics of the demographic variables are displayed in Table 1.

**Table 1:** Demographic characteristics.

Variable	Values	Frequency	Percentage
Gender	Boy	109	35.39%
	Girl	199	64.61%
Age	15	15	4.87%
	16	53	17.21%
	17	114	37.01%
	18	126	40.91%
Subjective social class	1	1	0.32%
	2	4	1.30%
	3	30	9.74%
	4	53	17.21%
	5	79	25.65%
	6	70	22.73%
	7	53	17.21%
	8	15	4.87%
	9	1	0.32%
	10	2	0.65%

### 3.2. Measures

#### 3.2.1. Myers-Briggs Type Indicator use

We adapted the Social Contagion-Conscious Behavioral Response (CBR) scale to measure MBTI use. The CBR was originally developed by Lopez et al. (2021) to measure astrology information use during the COVID-19 outbreak. The scale draws on 10 items to measure the three phases of MBTI use. These are the intake, output, and internalization phases (newly added), which describe the reading of information, analyzing one’s personality, and discussing the MBTI in SNSs, respectively. All items were measured on a five-point scale (1 = never, 5 = always). The average of the item scores demonstrated a respondent’s MBTI use level.

The CBR scale measured reported behavior regarding reading and the basic application of MBTI information. A deeper understanding of MBTI information was not covered. Therefore, we proposed three extra items to better comprehend a respondent’s MBTI use. An example is “the degree to which you are familiar with different MBTI dimensions and types.” We used the same five-point scale.

A factor analysis showed that these 13 items could be classified as one factor, reaching a high Cronbach’s alpha of 0.908.

#### 3.2.2. Level of susceptibility to the Barnum effect

We measured Barnum effect susceptibility by adapting and translating the scale by Lopez et al. (2021). Based on this scale, the Barnum effect susceptibility level to the MBTI was examined in three dimensions: personal accuracy rating (“the personality traits about my MBTI are accurate”), interpretation favorability rating (“the MBTI affirms my inner self”), and interpretation exclusivity rating (“the MBTI interpretations suit my characteristics more than they do other people”). The items were rated on a range of 1 to 5 (1 = strongly disagree, 5 = strongly agree). By computing the mean score, we acquired the participants’ susceptibility to the Barnum effect. The Cronbach’s alpha value was 0.875 in the sample.

#### 3.2.3. Ego identity

By referring to the mature and representative scale compiled by Balistreri et al. (1995), Zhou (2006) translated and revised an ego identity process scale for a Chinese context, which achieved good reliability and validity. We adopted it for the current research. The scale consists of 37 items, including 16 exploration and 21 commitment items. The scale is scored on a six-point Likert scale (1 = very non-conforming, 6 = very conforming), with four items being reverse scored. According to a factor analysis, we dropped three items that could not be associated with a specific factor (e.g., “I am confused about whether certain behaviors in love should occur”). The total Cronbach’s alpha value, including the exploration and commitment dimensions, was 0.997, exhibiting high reliability.

#### 3.2.4. Subjective well-being

The measurement of subjective well-being consisted of two widely applied scales: the Satisfaction with Life Scale (SWLS) and the Positive and Negative Affect Scale (PANAS). Both have existed in Chinese translations for a long time. The SWLS was first compiled by Diener et al. (1985) and consists of five items scored on a five-point Likert scale.

We used the Chinese edition of PANAS revised by Qiu et al. (2008). The scale contains a total of 14 emotion words (seven positive and seven negative), which are interspersed in the questionnaire. The items are ranked on a five-point scale and calculated separately by the positive or negative group. The Cronbach’s alpha values of the scale were 0.872 (complete SWLS), 0.879 (positive only), and 0.834 (negative only). We calculated subjective well-being by summing the standardized positive affect scores, negative affect scores, and life satisfaction scores (reverse coding was taken into account).

#### 3.2.5. Depression and anxiety

To measure the mental health of adolescents from both positive and negative aspects, we also investigated adolescent depression and anxiety levels. Based on the Symptom Checklist 90 (SCL-90; Derogatis and Unger, 2010), Derogatis and Melisaratos (1983) developed the Short Symptom Inventory (BSI). The scale consists of 53 items to identify nine factors: somatization, obsessive–compulsive symptoms, depression, anxiety, interpersonal sensitivity, hostility, phobic anxiety, paranoid ideation, and psychoticism. According to the purpose of this study, the depression and anxiety subscales, each consisting of six items, were used. A 5-point Likert scale was used for the items. The Cronbach’s alpha values of the two subscales were 0.829 (depression) and 0.840 (anxiety).

#### 3.2.6. Demographic information

At the end of the questionnaire, we included demographic variables such as gender, age, and subjective social class.

### 3.3. Data analysis

The data analyzes in the study were run using SPSS 26.0 and R4.2.2. Primarily, we took Harman’s single-factor test to exclude the problem of common method variance (CMV) for the variables that were measured by the self-report questionnaire. Twenty-six factors were extracted with characteristics greater than one. The largest accounted for 13.98% of the total variance (smaller than 40%). Therefore, there was no evidence of serious common method deviation in the current study. Then, we performed a correlation analysis between variables using SPSS 26.0. After that, R with Lavaan was applied to test the sequential mediation effect of the main analysis. In each mediation model, we selected a bias-corrected and accelerated bootstrap with 5,000 replicates and a 95% confidence interval.

## 4. Results

### 4.1. Descriptive statistics and correlation analysis

The means, standard deviations, and correlations among the variables are shown in Table 2. The results revealed that when controlling for demographic characteristics, MBTI use was positively correlated with Barnum effect susceptibility (*r* = 0.05, *p* < 0.001) and ego identity (*r* = 0.27, *p* < 0.001), and the latter two were also positively correlated with each other (*r* = 0.40, *p* < 0.001). MBTI use (*r* = 0.18, *p* < 0.01), Barnum effect susceptibility (*r* = 0.26, *p* < 0.001), and ego identity (*r* = 0.43, *p* < 0.001) all had significant positive relationships with subjective well-being. Ego identity was negatively correlated with depression (*r* = −0.26, *p* < 0.001) and anxiety (*r* = −0.15, *p* < 0.01). However, the relationship between MBTI use and depression (*r* = −0.02, *p* > 0.05) and anxiety (*r* = 0.11, *p* > 0.05) was not significant.

**Table 2:** Descriptive and bivariate correlations.

Variables	*M* ± SD	1	2	3	4	5	6
1. MBTI use	2.76 ± 0.81	–					
2. The Barnum effect	3.63 ± 0.51	0.53***	–				
3. Ego-identity	4.31 ± 0.46	0.27***	0.40***	–			
4. Subjective well-being	0 ± 2.25	0.18**	0.26***	0.43***	–		
5. Depression	2.26 ± 0.85	0.02	−0.13*	−0.26***	0.35***	–	
6. Anxiety	2.23 ± 0.84	0.11	−0.12*	−0.15*	−0.23***	0.71***	–

Control variables: gender; age; subjective social class *p < 0.05; **p < 0.01; ***p < 0.001.

### 4.2. Simple mediation analysis of ego identity

We constructed a mediation model to examine H1 and H2. The results indicated that the total effect of MBTI use on subjective well-being was significant [*β* = 0.45, 95% CI = (0.15, 0.76)], whereas the total effects on depression [*β* = 0.02, 95% CI = (−0.11, 0.14)] and anxiety [*β* = 0.11, 95% CI = (−0.01, 0.23)] was not. H1a was supported, while H1b and H1c were rejected.

The heterogeneity was also found in the direct effects of the three paths. The direct path from MBTI use to anxiety was significant [*β* = 0.17, 95% CI = (0.05, 0.28)]. However, the other two paths were insignificant [subjective well-being: *β* = 0.17, 95% CI = (−0.10, 0.46); depression: *β* = 0.10, 95% CI = (−0.03, 0.22)].

Then we looked into the simple mediation model from MBTI use on mental health *via* the mediator variable, ego identity. When the dependent variable was subjective well-being, the indirect effect was significant [β = 0.28, 95%CI = (0.16, 0.44)]. When we took depression and anxiety as the dependent variable respectively, the indirect effects were also significant [depression: *β* = −0.08, 95% CI = (−0.13, −0.04); anxiety: *β* = −0.05, 95% CI = (−0.10, −0.02)]. That is to say, using MBTI tools would add to the level of ego identity (supported H2) and, in turn, increase subjective well-being and decrease depression and anxiety (see Table 3). The results of the indirect effects supported our H3 (a-c). All of the above analysis controlled for gender, age, and subjective social class.

**Table 3:** Significant results of simple mediation models.

	Effect	Boot SE	*p*	Boot LLCI	Boot ULCI
MBTI use→ego identity→subjective well-being	0.28	0.07	*p* < 0.001	0.16	0.44
MBTI use→ego identity→depression	−0.08	0.02	*p* < 0.01	−0.13	−0.04
MBTI use→ego identity→anxiety	−0.05	0.02	*p* < 0.01	−0.10	−0.02

Bootstrap sample size = 5,000; SE, standard error; LL, low limit; UL, upper limit; CI, confidence interval.

### 4.3. Sequential mediation analysis of the Barnum effect and ego identity

**Figure 2:**
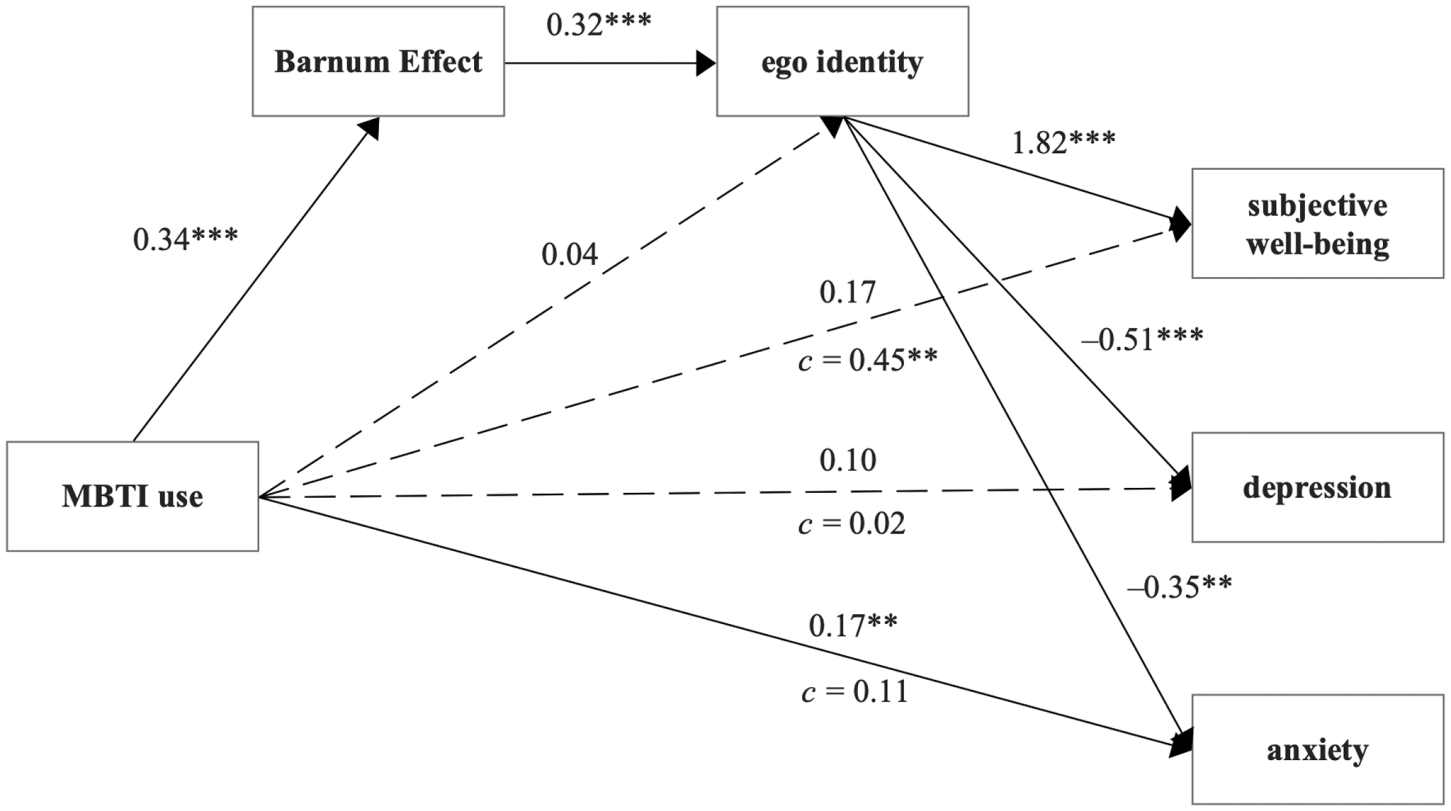
Sequential mediation models of Barnum effect and ego identity on the relationship between MBTI use and mental health (subjective well-being, depression, anxiety). *N* = 308, *p < 0.05, **p < 0.01, ***p < 0.001 (two-tailed test).

We then verify the sequential mediation model in H4. Controlling for gender, age, and subjective social class, we took MBTI use as the independent variable; Barnum effect susceptibility and ego identity were the mediators, and mental health was the dependent variable in the model. As predicted, the indirect path from MBTI use to subjective well-being through the Barnum effect and ego identity was significant [*β* = 0.20, 95% CI = (0.12, 0.31)]. The indirect effects of the sequential mediation model on depression and anxiety were also significant [depression: *β* = −0.06, 95% CI = (−0.10, −0.03); anxiety: *β* = −0.04, 95%CI = (−0.07, −0.01)]. When we added the Barnum effect, the relationship between MBTI use and ego identity was no longer significant, meaning that the Barnum effect completely mediated the effect of MBTI use on ego identity. Thus, the path consisting of the Barnum effect and ego identity together represents the potential mechanism between MBTI use and mental health. In conclusion, the data support H4 (a-c), suggesting that an elevated Barnum effect triggered by more MBTI use causes teenagers to have a higher ego identity and, as a result, contributes to an increase of subjective well-being and decrease of depression and anxiety (Table 4). The final model is displayed in Figure 2.

**Table 4:** Significant results of sequential mediation models.

	Effect	Boot SE	*p*	Boot LLCI	Boot ULCI
MBTI use→ego identity→subjective well-being	0.08	0.07	ns	−0.04	0.22
MBTI use→Barnum effect→ego identity→subjective well-being	0.20	0.05	*p* < 0.001	0.12	0.31
MBTI use→ego identity→depression	−0.02	0.02	ns	−0.07	0.01
MBTI use→Barnum effect→ego identity→depression	−0.06	0.02	*p* < 0.01	−0.10	−0.03
MBTI use→ego identity→anxiety	−0.02	0.01	ns	−0.05	0.01
MBTI use→Barnum effect→ego identity→anxiety	−0.04	0.02	*p* < 0.05	−0.07	−0.01

Bootstrap sample size = 5,000; SE, standard error; LL, low limit; UL, upper limit; CI, confidence interval.

## 5. Discussion

While the mechanisms of personality assessment and the Barnum effect have been well studied, little is known about the subsequent psychological effects of this path. The present study was conducted to determine the influence of the Barnum effect triggered by MBTI on adolescent ego identity and mental health (subjective well-being, depression, and anxiety). The results support that, for Chinese adolescents, MBTI use has a positive impact on adolescent mental health. However, the results showed heterogeneity due to the dependent variables. Furthermore, the Barnum effect and ego identity together act as an essential mediating path between MBTI use and mental health. Specifically, the personality feedback provided by the MBTI is somewhat vague and general, which motivates the Barnum effect among users. The Barnum effect then sequentially enhances the adolescent ego identity, elevates their sense of subjective well-being, and reduces the levels of depression and anxiety.

### 5.1. Heterogeneity in subjective well-being, depression and anxiety

Although we hypothesized a consistent effect of MBTI use on three aspects of mental health, our findings showed heterogeneity. Specifically, MBTI use affected subjective well-being (H1a was supported) but did not affect depression and anxiety (H1b and H1c were not supported). A longitudinal study by Janicke-Bowles et al. (2022) reports that posting and engaging with hedonic or inspiring Facebook content increased love and comparison to others (eudaimonic well-being) but had no influence on depression and anxiety. Considering that MBTI use is also related to entertainment and widespread “Internet meme” on SNSs, it is possible that well-being is more likely to be affected by MBTI use.

When looking into the direct effects in the sequential mediation model, MBTI use exerts a positive influence on anxiety, although its total effect is nonsignificant. This means when considering and controlling the Barnum effect and ego identity, MBTI use increases anxiety. The positive relationship was also reported by Clements (2020). He pointed out that habitual engagement in astrology requires individuals to make choices from the various identities and possibilities offered, leaving them cognitively disorientated and therefore threatening to anxiety. Such influence is suppressed, considering that the psychological process through the Barnum effect and ego identity can relieve anxiety.

In sum, MBTI use can positively influence well-being in the general context. Its role in negative aspects of mental health, such as depression and anxiety, is more complicated. It affects depression and anxiety through the Barnum effect and ego identity (see the discussion below).

### 5.2. The mediating role of ego identity

The simple mediation analysis supported our H2 and H3. Unscientific personality assessments are beneficial to adolescents in the identity-cognition stage, increasing their ego identity. These results are in line with previous empirical studies in astrology, which showed that the contract of astrology information is related to the promotion of self-concept (Lillqvist and Lindeman, 1998; Lopez et al., 2021). As mentioned above, ego identity can be understood as a maturity of self-concept (Guo and Che, 2004). This result extended the sources of self-perception theoretically. Among the information from the external world, apart from social comparison and reflexive evaluation (Brown, 2014), the usage of personality assessment can also influence ego identity through cognitive processes. There are several detailed possible explanations for this result. First of all, although the MBTI test is judged invalid and unreliable, some dimensions of MBTI are related to the Big Five dimensions, which indicates that the MBTI can interpret individuals’ personalities to some degree (Furnham, 1996). This result could also be due to the wealth of feedback from the MBTI results. Like astrology, the personality information of MBTI types ranges from strengths and weaknesses, romantic relationships, and friendships to career paths. Similarly, ego identity is defined and measured from these aspects, including exploration and commitment to hobbies, romantic relationships, careers, etc. These factors may explain the correlation between MBTI use and ego identity. As mentioned in the literature review, ego identity is crucial for mental health, and the data we collected reflected those ideas (Hofer et al., 2007; Waterman, 2007; Cakir, 2014). Specifically, the current study found that high ego identity inspired by MBTI also benefits adolescent mental health, such as increasing subjective well-being and decreasing depression and anxiety. To conclude, taking the MBTI assessment as an example, this study demonstrates that low-valid personality assessment can help enhance ego identity and, consequently, maintain adolescent mental health.

### 5.3. The sequential mediating role of the Barnum effect and ego identity

The few studies on the relationship between personality assessment and ego identity lack an in-depth analysis of the underlying mechanism. Through a review of the literature, we assumed a possible mediating role of the Barnum effect. The present results suggest that the more frequently adolescents use the MBTI, the more they perceive these explanations to be accurate and distinct. It supports previous studies on the correlation between the Barnum effect and low-effective personality assessments, such as the MBTI, the Rorschach test, and astrology (Dickson and Kelly, 1985; Furnham and Schofield, 1987). In addition, the current work strives to test the possibility proposed in previous studies. Both Lillqvist and Lindeman (1998) and Lopez et al. (2021) have mentioned the potential of the Barnum effect in explaining the association between the use of personality assessment and self-concept. The data presented here demonstrate its existence. This may be related to the following reasons. Firstly, according to the self-validation theory (Swann and Read, 1981) and positive illusion theory (Taylor and Brown, 1999), people tend to organize self-related information in order to strengthen their self-concept automatically. Secondly, the manipulation definition of ego identity consists of exploration and commitment processes. That is, the more susceptible adolescents are to the Barnum effect, the more receptive they are to a wealth of personality explanations, and the higher their level of self-exploration. Similarly, susceptibility to the Barnum effect also affects their recognition of Barnum feedback, further altering their level of self-commitment. Taken together, this means that the Barnum effect may influence adolescent ego identity.

Turning to the sequential mediation model, the data in this study support our H4. That is, frequent MBTI use by adolescents will stimulate the Barnum effect, which then enhances their ego identity and, ultimately, increases their subjective well-being levels and also reduces their anxiety and depression levels. These findings suggest that low-validity personality assessments may help maintain adolescent mental health. Apart from obtaining social connectivity and entertainment from personality assessments (Allum, 2011; Lu et al., 2020), individuals can also acquire positive psychological benefits through the “Barnum effect ⟶ ego identity” mechanism. Hopefully, it may be easily triggered by reading and assessing ample personality feedback involving identity traits, life attitudes, or relationship advice.

### 5.4. Implications

#### 5.4.1. Theoretical implications

The present research discusses the positive psychological implications of personality assessments, as exemplified by the MBTI, and examines the underlying mechanisms. First, we conducted empirical research based on previous findings regarding personality assessment use and the self. The Barnum effect was introduced as a mediating variable to explain the pathway between personality assessment use and adolescent ego identity. Second, because ego identity is crucial to adolescent mental health, we further explored whether personality assessment would increase adolescents’ subjective well-being and decrease their depression and anxiety after enhancing their ego identity. The results support our hypotheses.

This research provided an alternative perspective from which to study the Barnum effect. We examed whether the MBTI can trigger the Barnum effect in individuals as with other assessment tools and then investigated its subsequent psychological effects on individuals. The empirical findings in the current study preliminarily demonstrate the psychological significance of the Barnum effect and enrich the theory. It also lays the groundwork for future research on the psychological consequences and social implications of the Barnum effect.

The current study also contributes to the existing research literature on adolescent mental health. By taking a low-validity personality assessment as a predictor of adolescent well-being, we found a new potential path for maintaining adolescent mental health. The sequential mediation model demonstrated a link between MBTI use, the Barnum effect, ego identity, and mental health. These findings open up more possibilities for future research on adolescent mental health.

Moreover, most existing studies were conducted in Western countries, which lack external validity in other contexts. These findings validate the consequential impact of personality assessment and the Barnum effect in a Chinese context, making them a useful contribution to the literature.

#### 5.4.2. Empirical implications

The findings of this study also have significant implications for future practice.

Given that low-validity personality assessments are widely recognized and believed to be effective among laypeople, the current study attempts to investigate their social impact. These positive results remind us to hold a deeper insight into the application of MBTI and other personality assessments. In addition to test the reliability and validity, we should also value the contribution of personality assessments in constructing ego identity, alleviating anxiety and depression, and enhancing well-being.

In addition, this study provides a theoretical pathway for a new approach to adolescent mental health interventions. As noted above, the adolescent population rarely seeks professional help when confronted with psychological problems, but will actively explore some self-help services. The present findings suggest an original but simple idea. By manipulating the personality information they receive, we can effectively stimulate the Barnum effect, expand the dimensions of their self-perceptions, and further increase the adolescents’ ego identity and subjective well-being. This is a cost-effective intervention compared to the relatively higher investment involved with professional counseling on one end and the low effectiveness of arbitrary information searches on the other.

### 5.5. Limitations and future works

Notwithstanding the contributions of the current study, there are some limitations. First, the cross-sectional data used here impact the causality among the covered variables. Future longitudinal designs are encouraged to retest the mediation model in the current study.

This study lacked the heterogeneity analysis of the pathway mechanism. For example, the present research measured the variable described as “MBTI use” at a rather high level. We integrated various MBTI usage behaviors into a general variable and found a primary relationship between it and other variables. Referring to extant research on the use of SNSs, more detailed measurements have been applied, such as positive and negative use. The results have indicated that diverse use behaviors have significantly different effects on users’ psychology (Meier and Reinecke, 2021). In the present study, we also found that MBTI has different effects on depression, anxiety, and well-being. Although we proposed possible explanations, further investigation is necessary. In addition, individual factors such as gender and personality were found to be associated with the Barnum effect (Fichten and Sunerton, 1983; Piper-Terry and Downey, 1998). Future work can explore the heterogeneity of the pathway by looking into individual differences.

Furthermore, the generalizability of these results is subject to certain limitations. Future research could move beyond the context of the MBTI and use other personality assessments or more general descriptive personality texts to explore the feasibility of a “textual features ⟶ Barnum effect ⟶ ego identity ⟶ well-being” pathway. As mentioned above, an exploration in this field would greatly help to test the causality of the current results and simplify the text and processing of mental health intervention for high school students, which may have higher practical value in the future.

## Data availability statement

The raw data supporting the conclusions of this article will be made available by the authors, without undue reservation.

## Ethics statement

This study involving human participants were reviewed and approved by Nanjing University. Written informed consent from the participants’ legal guardian/next of kin was not required to participate in this study in accordance with the national legislation and the institutional requirements.

## Author contributions

JH and Y-XZ contributed to the conception and design of the study. JH performed the statistical analysis and wrote the first draft of the manuscript. Y-XZ developed the general framework of the manuscript and reviewed the manuscript. All authors contributed to the manuscript revision, read, and approved the submitted version.

## Funding

The work was supported by the Fundamental Research Funds for the Central Universities (No. 011014370119).

## Conflict of interest

The authors declare that the research was conducted in the absence of any commercial or financial relationships that could be construed as a potential conflict of interest.

## Publisher’s note

All claims expressed in this article are solely those of the authors and do not necessarily represent those of their affiliated organizations, or those of the publisher, the editors and the reviewers. Any product that may be evaluated in this article, or claim that may be made by its manufacturer, is not guaranteed or endorsed by the publisher.

## Footnotes

^1^ www.wjx.com
